# Systematic Review on Mentalization as Key Factor in Psychotherapy

**DOI:** 10.3390/ijerph18179161

**Published:** 2021-08-31

**Authors:** Jonas Lüdemann, Sven Rabung, Sylke Andreas

**Affiliations:** Institute of Psychology, University of Klagenfurt, Universitätsstr. 65-67, 9020 Klagenfurt am Woerthersee, Austria; Sven.Rabung@aau.at (S.R.); Sylke.Andreas@aau.at (S.A.)

**Keywords:** systematic review, mentalization, reflective functioning, psychotherapy, outcome

## Abstract

Background: Mentalization processes seem to be of high relevance for social learning and seem important in all psychotherapies. The exact role of mentalization processes in psychotherapy is still unknown. The aim of the present systematic review is to investigate whether mentalization is related to the therapeutic outcome and, if so, whether it has a moderating, mediative, or predictive function. Method: A systematic review with an electronic database search was conducted. A total of 2567 records were identified, and 10 studies were included in the final synthesis. Results: Psychotherapy research is still in an initial phase of examining and understanding the impact of mentalization on psychotherapy outcome. The small number of studies and the executed study designs and statistical analyses indicate the possible role that mentalization has in psychotherapy. Conclusion: Generally, strongly elaborated study designs are needed to identify the role of mentalization in psychotherapy. Mentalization seems to be differently represented in differential treatment approaches. Nevertheless, it should be noted that the patient’s mentalizing capacity seems to be relevant to the psychotherapy process. Psychotherapies should be adapted to this.

## 1. Introduction

In recent years, the new concept of Mentalization Based Psychotherapy (MBT) has become increasingly popular in psychodynamic psychotherapy and research [1,2,3,4,5]. Mentalizing is defined as the capacity to understand other people’s intentional or inner mental states while taking into account one’s own intentional states (e.g., beliefs, thoughts, feelings, desires, goals [6,7].

The mentalizing approach was developed by Fonagy, Steele, and Steele within the London Parent–Child project [6]. The authors observed that a child’s secure attachment is not only dependent on the mother’s attachment security [6], but rather results from the mother’s insight to acquire a psychological understanding of her early childhood relationship with her parents [8]. In regard to their predominantly positive attachment experiences and the associated secure attachment representations, 79% of these mothers also had securely attached children compared to the 28% for mothers with an insecure attachment style. Hence, the assumption could be confirmed that those mothers who reported more negative childhood experiences during the interview were nevertheless able to provide coherent statements and coping strategies due to their high reflexive competence [8].

The empirical findings of this study, which was conducted Fonagy et al., believe that there must be a connection between early attachment experiences [8], self-representation, and affect regulation even in patients with severe personality disorders [4]. Empirical studies have shown that patients with severe personality disorders have lower mentalizing capacities than normal people [9,10,11,12,13]. In the meantime, this has also been shown for patients with other mental illnesses such as depression [14,15,16,17,18] or eating disorders [16].

As neuropsychological research shows, mentalization can appear in four different dimensions [19,20]. The first dimension refers to automatic versus controlled mentalizing. Controlled mentalization is a serial and relatively slow process that is usually expressed verbally and requires reflection. In contrast, automatic mentalization is a much faster process requiring only a little attention, intention, awareness, and effort [21]. In this dimension, mentalization problems arise when individuals insist exclusively on automatic assumptions about the mental states of themselves or others. Difficulties also arise when the situation becomes difficult for the person to adequately apply these automatic assumptions. The second dimension includes the ability to mentalize oneself or the mental states of others (self- versus other-mentalizing). This means the ability to understand one’s own inner mental states of beliefs, desires, needs, and those of others. Mentalization can also refer to drawing conclusions based on external cues (e.g., facial expressions and gestures). However, it can also refer to understanding someone’s internal experience from what a person knows about the person and the situation they find themselves in (internal versus external mentalizing). The fourth dimension refers to distinguishing whether the person understands mental states more cognitively or affectively (cognitive versus affective mentalizing). Cognitive mentalization involves the ability to name, perceive, and find reasons for inner states, while affective mentalization involves the ability to understand the feelings of inner states [21]. The dimensions described above are categories in which mentalization can take place. Disturbances of the mentalization ability are present if one pole of a dimension (e.g., cognitive mentalization) is too strongly pronounced [21].

MBT was originally developed by Bateman and Fonagy [3] for borderline patients. The objective of MBT is to improve the mentalization capacity of patients with severe personality disorders throughout the various dimensions [21]. Allen, Fonagy, and Bateman [22] postulate that the concept of mentalization is represented in all forms of psychotherapy and that the facilitation of mentalization makes psychotherapies effective [23]. Recently, Fonagy et al. [24] have expanded the hypothesis from that of an evolutionary and developmental point of view, indicating that improvement in mentalization is needed to maximize the benefits from social experiences to get along better in the social world. In turn, this is only possible in a therapeutic relationship that is perceived as secure. Fonagy et al. [24] postulate that therapeutic change can only occur in a secure therapeutic relationship that they refer to as epistemic trust. By epistemic trust, we mean the willingness to accept social messages and messages relevant and helpful to the person within the therapeutic relationship [24]. Epistemic trust provides the basis for social learning and enables individuals to benefit from their own social environment. It is believed that most mental disorders are due to epistemic mistrust, where the individual is unable to benefit from social conversations and interactions. As a result, these individuals experience difficulties updating their own expectations and beliefs and adapting to changing situations. Epistemic trust allows the individual to adapt his social imagination to the prevailing social reality through mentalization [24]. Therefore, mentalization probably has a more mediating function. A patient who has mentalization problems will probably also misinterpret the ostensive cues so that no epistemic trust can be built [24]. That implies that mentalization processes should be of great importance in any evidence-based psychotherapy [25].

### 1.1. Operationalization of Mentalization

The assessment of mentalization or reflective functioning, the operationalization of mentalization, can be conducted via interview assessment, questionnaires, or visual tests. Up to this point, a variety of instruments that can measure different areas or dimensions of mentalization capacity are available (e.g., the cognitive-affective dimension, [26]). The most widespread expert assessment method is the Reflective Functioning Scale (RFS) [26], which is collected based on adult attachment interviews (AAI) [26]). Based on the narratives in the AAI, the mental states of the subjects are assessed on a scale from −1 (rejected reflective functioning) to 9 (very high reflective functioning). The RFS is a reliable and valid instrument that requires the raters to be specifically trained in its administration. Slade and Slade et al. combined the RFS with the Parent Development Interview (PDI) [26], hence developing the Parental Reflective Functioning Coding System (PRF-CS) [26]. The RFS based on the PDI is a reliable and valid method to measure the reflective functioning of parents regarding their relationship to their children, their parenthood, and the perception of their children [26]. Since the AAI is a time-consuming procedure, there has been an increased effort to directly measure reflectivity in specific mental disorders in recent years. One of these instruments is the Depression-specific Reflective Functioning Interview (DSRF) [26], which has shown very good results in initial reliability and validity studies [26]. Considering that the AAI has an average interview duration of 1 to 2 h [26], Rudden et al. [27] developed a specific interview, the Brief Reflective Functioning Interview (BRFI; [27]). The questions in the BRFI are intended to provoke reflections on attachment experiences and were developed based on the AAI. The assessment of reflective functioning via BRFI follows the same principle as for the AAI, albeit an average value is calculated for all ten questions. The BRFI thus represents a useful, economical, reliable, and valid method for measuring reflective functioning.

One recently developed self-assessment instrument is the Reflective Functioning Questionnaire (RFQ) by Fonagy et al. [28] The instrument uses 12 items on 2 subscales, measuring security and insecurity in reflective functioning. Three studies have demonstrated both reliability and validation in clinical samples, whereas the scale of insecurity did not show satisfactory results in a non-clinical sample [26]. Analogous to the PDI, the Parental Reflective Functioning Questionnaire (PRFQ; [29]) was developed as a self-assessment measure. The PRFQ measures parental reflective functioning on three dimensions: interest and curiosity in the mental states of the infant, pre-mentalizing modes, and certainty about the mental states of the infant. A first study demonstrated reliability and validity [30]. The methods presented so far are all based on the AAI and the RF scale developed by Fonagy et al. [28]. Further methods for measuring reflective functioning, primarily focusing on emotional perception, can be found in Luyten et al. [4].

Mentalization-based psychotherapy has gained increasing importance in recent years. Moreover, mentalization processes seem to be of high relevance for social learning and could thus be vital for all psychotherapies. However, it is unknown what role mentalization processes exactly have in psychotherapy. Although studies have been conducted on mentalization processes in psychotherapy (i.e., [31,32]), this is still the case. In this regard, the existing evidence has not yet been synthesized. Thus, a systematic review of studies examining the relationship of mentalizing processes in the context of psychotherapy outcome-relevant processes can provide new insights into mentalization processes for psychotherapy research and practice. Therefore, the aim of the present systematic review is to investigate whether mentalization is related to the therapeutic outcome and, if so, whether it has a moderating, mediative, or predictive function.

### 1.2. Concepts of Psychotherapy Process Factors

It is necessary to differentiate concepts describing how variables may be associated with psychotherapy outcome.

A moderator is a statistical baseline variable that has an interactive effect on the outcome. Moderators suggest different directions or magnitudes in the relationship between an independent and dependent variable in specific subgroups. Thus, the influence of an independent variable on a dependent outcome variable is moderated by a third variable [33,34,35].

A mediator is a statistical variable intervening in the relationship between the dependent variable (outcome) and the independent variable (treatment). This means that the independent variable is not only related to outcome, but to the proposed mediator variable too, which again is related to outcome. Thus, the relationship between treatment and outcome becomes smaller after controlling for the mediator effect. Even though mediators alone cannot explain the precise process of change, they may point to possible mechanisms without necessarily being part of it [33].

A predictor is a statistical baseline variable (not mediator) that predicts outcome independent of subgroups (not moderator) [35].

As these concepts provide a basis for examining the association of a variable with psychotherapy outcome, the treatment must be proven effective in changing the outcome before any further investigation [35]. Moreover, it is impossible to distinguish between predictors and moderators or mediators of treatment effects without a comparison group [35].

Considering these concepts, the following research questions were investigated in this systematic review: (I) Is mentalization related to psychotherapeutic treatment outcome? (II) If mentalizing is related to the outcome of psychotherapeutic treatment, does it act as a moderating, mediating, or predictive factor?

## 2. Method

### 2.1. Literature Search and Study Selection

An electronic database search was conducted using the databases: Ovid MEDLINE(R); Epub Ahead of Print, In-Process & Other Non-Indexed Citations, Daily and Versions(R) 1946 to 10 September 2019; and PsycINFO 1806 to September Week 2 2019. In order to identify relevant papers, the following search strategy was executed: “((“reflective function*” or mentaliz* or mentalis*) and (psychother* or therap* or treatment*)).mp.”. Following the electronic database search, two raters independently screened the titles and abstracts considering the inclusion and exclusion criteria. There was no blinding for journal titles, study authors, or institutions. In a second step, full-text screenings were conducted for those reports that the screeners had been uncertain about earlier. Finally, the reference lists of all of the included studies were reviewed. Any publications citing these studies were identified and were checked for eligible reports. Disagreement was resolved through discussion.

### 2.2. Inclusion Criteria

Empirical studies (minimum evidence level III [36]) examining the impact of mentalization on the process and outcome of psychotherapy were considered for this systematic review. Studies published prior to 1991 were not considered, as the concept of mentalization was introduced during that year [37]. Participants in the study had to be 18 to 65-year-old patients with a DSM/ICD diagnosis in comprehensive psychotherapy (includes individual psychotherapy as a substantial part of the intervention; additional group therapy may or may not be included; at least three months duration). Thus, the psychotherapeutic intervention had to have evidence-based psychotherapy to a substantial part, lasting for at least twelve sessions or three months. Additional group therapy could be included, but studies were excluded if the central intervention was group therapy or art therapy. The use of standardized outcome instruments was required. Additionally, the assessment of mentalizing capacity had to be reported [4]. Studies had to be published in peer-reviewed journals.

### 2.3. Data Extraction

Subsequent to the screening process, the following information was extracted from the included studies: publication (authors, year of first publication, region), type of study, sample (*n*, diagnosis, age, gender), treatment context (therapy, treatment duration, number of therapists), operationalization of mentalization, function of mentalization (predictor/moderator, mediator/outcome), outcome instrument, result (pre/post values: mean values (M), and standard deviations (SD) if applicable; simple summary data for each intervention group at least).

## 3. Results

### 3.1. Study Selection

**Figure 1:**
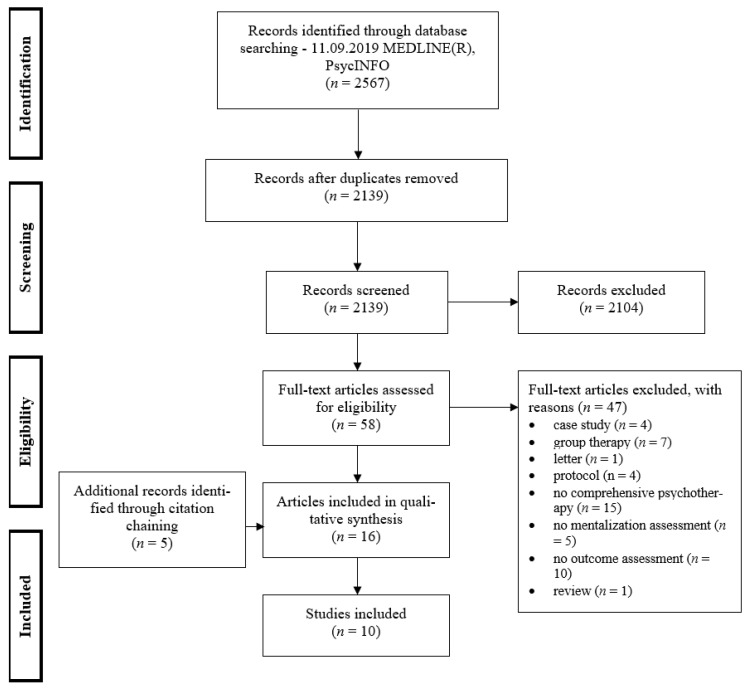
PRISMA flow diagram (Moher et al. [38]).

The electronic database search was conducted on 11 September 2019. In total, 2139 potentially relevant articles were identified, and finally, 10 studies fulfilling the eligibility criteria were included. The agreement of the two raters was 99.25%. Differences or unclear allocations (k = 16) could be resolved by consensus. The study selection process is illustrated in Figure 1.

### 3.2. Study Characteristics

An overview of the included studies and their characteristics is summarized in Table 1. The studies were conducted in geographical Europe and the USA. Half of the included studies (originally) were RCTs, and the other studies followed a pre–post design. The sample size ranged between *n* = 20 and *n* = 138 patients, respectively, *n* = 2 and *n* = 67 therapists. Most of the studies (k = 4) included patients with a major depressive disorder diagnosis, while three studies included patients with a personality disorder diagnosis. The rest of the studies examined patients with mixed diagnoses (k = 3; with mainly depressive disorders). Treatments were mainly psychoanalytic/psychodynamic, though cognitive behavioral therapy (CBT) and other treatment approaches were examined. The mean treatment duration ranged between 14 and 366 sessions. In all of the included studies, a pre–post improvement of outcome was measured (significant improvement in 80% of the studies).

### 3.3. The Mentalization Variable in the Psychotherapy Process

Most of the included studies used the RFS for the assessment of mentalization. Further measurements that were used were the Computerized Text Analysis measure of Reflective Functioning (CRF; [55]), the DSRF [26], and the Panic-Specific Reflective Functioning (PSRF; [27]). The original application of the RFS on the AAI was executed in four studies. Three studies used a short form of the AAI, and two applied the RFS on transcribed psychotherapy sessions. CRF was also applied during psychotherapy sessions, DSRF on an AAI short form, and the PSRF was used with an associated interview. In two studies, mentalization was only measured at baseline or within the first week of treatment, whereas the other studies (80%) had at least one other second measurement point to assess the capacity of mentalization.

### 3.4. Mentalization as Predictor

Five studies investigated the possible role of mentalization as a predictor of psychotherapy outcome via the RFS. In the study by Antonsen et al. [39], linear mixed modeling (LMM) analyses with the parameters for RFS and time were without significant longitudinal effects predicting the improvement of outcome variables (with the GSI for symptom distress) [39]. The six-year follow-up findings were in line with the 3-year follow-up analysis of the same sample by Gullestad et al. [40]. Bressi et al. [45] reported a significant HAM-D improvement predicted by the RFS score at baseline (β = 0.75, *p* = 0.025), explaining 13.8% of the variance in the HAM-D change [45]. Ekeblad et al. [46] reported a significant prediction of the initial RFS score on BDI-II improvement over 14 sessions with a medium-sized effect of β = 0.35 (standardized regression coefficient). In a three-month inpatient psychotherapy, RFS also significantly predicted overall improvement (*r* = −0.37 (*p* < 0.05) for RFS and the GSI at termination, partialling out the effect of GSI at onset and the effect of the overall structural level according to the operationalized psychodynamic diagnostics (OPD), as examined by Müller et al. [53]. Mixed results were found by Taubner et al. [54]. For a period of 36 months, Taubner et al. [54] found a significant BDI improvement predicted by the RFS score at baseline (*r* = −0.48, *p* < 0.05), explaining 23.04% of the variance in BDI-change, while there was no significant effect on the initial RFS score on GSI change (r = −0.318, *p* = 0.17) [54]. Previous examinations of the investigated sample after 8 months and after 15 months of treatment could not find significant effects of RFS scores on BDI/GSI scores and changes [17,18].

In two studies, a different measurement of mentalization method was used to investigate the possible role as a predictor. Besides the RFS, Ekeblad et al. [46] also applied the DSRF [26,46]. They found a significant prediction on BDI-II improvement over 14 sessions (β = 0.41). Boldrini et al. [44] examined therapy sessions with the CRF and found that CRF significantly predicted the final personality health index (PHI; [57]) and GAF scores as well as the pre–post changes in these measures [44]. However, for the analysis, Boldrini et al. [44] mixed initial scores with scores after one month of treatment; thus, the results do not represent a bona fide predictor analysis [44].

### 3.5. Mentalization as Moderator

The examination of mentalization as a possible moderator was only reported by Antonsen et al. [39]. Regarding the treatment type, Antonsen et al. found significant moderator effects of RFS on GSI depending on the patients’ mentalization capacity [39]. Patients with a medium RFS at baseline (RFS of 3 or above) had smaller effect sizes on all of the outcome variables in the outpatient treatment than patients in the control treatment. The comparison in patients with a low RFS at baseline (RFS below 3), on the other hand, resulted in greater effect sizes in outpatient treatment.

### 3.6. Mentalization as Mediator

None of the included studies conducted a state-of-the-art mediator analysis examining an overall treatment effect (outcome change) with a control condition additionally examining the treatment effect on mentalization (change), and if applicable, the effect of the change in the mentalization capacity on outcome change. While a possible mediating effect of mentalization in the psychotherapeutic process was not examined, half of the included studies investigated the change of mentalization in treatment and its impact on the outcome. 

### 3.7. Mentalization Change

Fischer-Kern et al. [47] examined the change in mentalization in two treatment arms (transference-focused psychotherapy, TCP, and treatment by experienced community therapists, ECP). They found significant improvements in mentalization after one year of TFP, but no significant improvements in ECP. They could also show a correlation of improved mentalization with better outcome scores, but they did not examine the directional causation of mentalization change on outcome change.

Barber et al. [42] examined early mentalization change with the RFS and PSRF in two comprehensive psychotherapy approaches (CBT and Panic-Focused Psychodynamic Psychotherapy; PFPP). They examined the association of mentalization change from intake to week five of the treatment on the subsequent change in panic severity until the termination of treatment. For both conditions, Barber et al. [42] found no improvement in the RFS. Additionally, the test on the association of early RFS change on change in outcome (PDSS) was not significant in both treatments. The application of the panic specific mentalization instrument PSRF led to different results. Early PSRF change was significant in PFPP but not in the CBT condition. Nevertheless, early PSRF change was associated with a significantly greater change in outcome (PDSS) in both interventions and was (not significantly) stronger for CBT. Due to the missing control condition, it is unclear if the change of symptom specific mentalization was caused by (a specific) treatment or not. Besides reporting that there is no early change in RFS (without a significant effect on outcome change) and only an early change in PSRF in PFPP, the results of Barber et al. [42] indicate that the magnitude of early mentalization change has a higher impact on outcome change than the mean probability of occurrence of mentalization change.

In the study by Taubner et al. [54] the RFS change examination is only reported for the therapy condition and not for the control condition (baseline RFS scores were reported for both conditions in Taubner et al. [18]). Due to the missing control group, it remains unclear if the RFS change is an effect of the psychotherapeutic treatment. Taubner et al. [54] found a significant improvement in the RFS from baseline to the second assessment 24 months later (with a medium effect of d = 0.61, *p* = 0.039). Further, Taubner et al. [54] examined a possible correlation between the change of RFS and at symptomatic change between baseline and the 36-month assessment. They found neither a significant correlation between RFS change and BDI change nor between RFS change and GSI change. It is still a question of whether the non-significant correlations are explicable based on the treatment effect. Thus, the results only allow very limited interpretations regarding the role of mentalization change in treatment.

In their first study, Karlsson and Kermott [50] found a significant RFS (measured on session transcripts) decrease from session 4 to session 12 for IPT, while they did not find any significant RFS change for CBT. In a post hoc analysis, they examined significant correlates between high versus low RFS with PQS items. A subsequent analysis with the relevant PQS items and the outcome showed that, in general, the correlation of PQS items with high RFS correlated with good outcome scores and that PQS items correlating with low RFS correlated with poor outcome. No intervention comparison was reported for the post hoc analysis. Based on that and the missing analysis of the direct impact of mentalization on the outcome, the influence of mentalization change in treatment can scarcely be evaluated.

The second study by Karlsson and Kermott [50] was an analogous examination of a BPDT treatment (sessions 1, 5, and 14). They found no significant RFS change during treatment. The same post hoc analysis was executed as in their first study, and they found correlations between high versus low RFS and good versus poor outcome. Nevertheless, many correlations did not achieve significance due to low statistical power, as reported by Karlsson and Kermott [50]. The interpretation of the results is comparably limited, as is also the case for their first study.

As mentioned in the section on the predicting role of mentalization above, Boldrini et al. [44] examined the prediction of an early period CRF score (which was a mean cumulation of the first four sessions of the treatment and four sessions after one month of treatment) instead of the prediction of an initial CRF score. Boldrini et al. [44] examined sessions from three treatment phases (early, middle, late) and found no significant CRF change. An analysis of CRF course impact on outcome was not reported.

## 4. Discussion

The role of mentalization as a predictor is not clear in terms of longitudinal effects—Antonsen et al. [39] seem to negate it, while the results of Taubner et al. [18,54] suggest a longitudinal effect, and the results of Boldrini et al. [44] are not comparable due to the different design of the data-analysis. The effect seems to be more consistent in shorter investigations [45,46,53], all being in favor of a positive prediction of psychotherapy outcome by the initial capacity of mentalization. 

The role of mentalization as a moderator was only examined by Antonsen et al. [39]. The study could be an indication that patients with different levels of mentalization require different treatment types.

For the role of mentalization as a mediator, no state-of-the-art mediator analysis was examined. Yet, half of the included studies give an indication for further research on that topic. Regarding that, it is interesting whether mentalization changes during the psychotherapy process for the examination of mentalization as a mediator (Table 2). 

The capacity of mentalization may change in the psychotherapy process but does not always change in psychotherapy. Two studies found a significant pre–post increase of RFS [47,54], one found a significant increase of PSRF (while RFS did not change significantly in the same study) [42], one examined a significant RFS change in the follow-up assessment (while RFS did not change significantly in the same study) [45], and one found a significant decrease of RFS (study 1) [50]. Two studies reported no significant change in mentalization (study 2) [44,50], and three studies did not examine or report a change of mentalization [39,46,53]. The differential results reveal that the change of mentalization in the psychotherapy process is not consistent. Studies examining mentalization change with at least two treatment conditions indicate that mentalization change differs depending on treatment (for RFS: [47,50]; for PSRF: [42]). The link between mentalization change and outcome change remains unclear regarding this point. While Barber et al. [42] and Taubner et al. [54] did not find an association of RFS change with outcome change, Fischer-Kern et al. [47] did, and Barber et al. [42] found an association for PSRF.

Instead of an explicit distinction, the results indicate that mentalization may hold several roles in the psychotherapy process at the same time. One possible explanation may be found in the conceptualization of mentalization. This integrates different dimensions (e.g., self-other, cognitive-affective, internal-external, automatic-controlled [19,20]), which may but do not have to simultaneously emerge and (differently) affect the psychotherapy process.

The review of the current status of research on the role of mentalization in the psychotherapy process reveals that psychotherapy research is still in an initial phase of examining and understanding the impact of mentalization on psychotherapy outcome. Besides the small number of studies examining the associations between mentalization (change) and outcome (change), the executed study designs and statistical analyses indicate that, at maximum, the systematic summary of the results only permits indications of the possible role that mentalization has in psychotherapy.

The data availability does not allow for more than one to report observations made in the systematic rework of the included studies. One of these observations is that the in the studies that lasted for one year, changes in mentalization were found. The changes in mentalization may take place but not before a particular time in treatment. Nevertheless, the included studies supporting the assumption of predictive effects indicate that attention should also be paid to the initial capacity of mentalization. However, these significant results are not definite in terms of the exact role of mentalization due to missing control conditions.

## 5. Conclusions

Overall, the included studies cannot directly support the postulate by Allen et al. [22], who stated that mentalization is represented in all forms of psychotherapy and makes them effective by means of facilitation of mentalization [22]. While it remains unclear if mentalization improves during psychotherapy, mentalization seems to be represented differently in various treatment approaches. Surprisingly, MBT was not examined in any of the studies. Therefore, the role of mentalization is still not explicit for this mentalization specific treatment.

All assumptions and postulates on the mediating role of mentalization remain without empirical support. Future examinations need complex and comprehensive study designs to control for the real effects of mediation. A more (time) efficient operationalization of mentalization is needed to trace mentalization in the psychotherapy process. For example, this is necessary to demonstrate a timeline of change for the proposed mediator variable occurring before outcome changes [34]. For this reason, instruments such as self-report measures or computer text analysis represent suitable approaches. However, these measures need to be further elaborated, so the time-consuming rating measures still need to be utilized momentarily. Furthermore, at the current state of mentalization research, the comparability of the different instruments should be repeatedly verified despite the existing construct validation. In this regard, the advantages and disadvantages for the use and development of disorder-specific measures versus a general operationalization of mentalization could also be further explored. Generally, strongly elaborated study designs are needed to identify paths of clinical change mechanisms that have an impact on mentalization or that are influenced by mentalization (change) [34]. By using control groups, future studies may further examine the role of mentalization by focusing more on different treatment approaches and patient groups.

While there is still much research needed to empirically understand and define the role of mentalization in the psychotherapy process, the results of this systematic review have at least one implication for practice: the patient’s mentalizing capacity matters, and the psychotherapeutic treatment should (also) be adapted to this.

## 6. Limitations

This review critically investigated the current psychotherapy research on the role of mentalization in the process of outcome improvement. One limitation was that the research question itself. The review examined how mentalization explains outcome change; thus, it did not examine variables that have a positive, a negative, or a compensatory effect on mentalization itself. Because of that, it remains unclear as to what determines mentalization change. The narrow inclusion criteria excluded studies examining the process of mentalization without investigating the association with psychotherapy outcome. Those studies should be taken into account for an update of this review to establish profound evidence on possible mentalization impact. 

As in any systematic review, this systematic review is restricted by the time period of the literature search and the study selection process. To ensure that the detections are not outdated, an update of the literature search and study selection process was conducted by two reviewers in August 2021. The electronic database search was repeated for the period after the original database search (k = 724 record identified), and publications from this period that cited the included studies were also screened. Finally, one additional study fulfilled the eligibility criteria [58]. This study investigated the relationship between mentalization, psychotherapeutic alliance, and treatment outcome in psychoanalytic psychotherapy (24 months) and CBT (5 months) in bulimia nervosa patients. The *n* = 70 patients were randomized, and the mentalization and treatment outcomes were assessed at three time points (baseline, after 5 months, after 24 months). Mentalization was measured with the RFS. No correlation between the baseline RFS score and outcome could be found. It was found that the RFS scores increased more in patients in the psychoanalytic psychotherapy condition than in the CBT condition. A significant relationship between RFS change and symptom change in the psychoanalytic psychotherapy condition can be seen as a further indication of the role of mentalization as a mediator in the psychotherapy process. Furthermore, the treatment type and duration had an impact on how mentalization changed and affected the treatment outcome [58]. The results fit with the findings of this systematic review.

Similar to the included studies, the review itself could not grasp the complexity of mentalization in the psychotherapy process. In future examinations, possible predictors, moderators, and mediators of mentalization should also be considered. Step by step, an empirically based mentalization process model could be derived from this.

## Figures and Tables

**Table 1:** Included studies.

	**Antonsen 2016**	**Barber 2020**	**Boldrini 2018**	**Bressi 2016**	**Ekeblad 2016**
Publication	[39,40,41]	[42,43]	[44]	[45]	[46]
		(NCT00353470)			
Country	Norway	USA	USA	Italy	Sweden
Study type	RCT	RCT	Pre–post ^n,d^	Pre–post ^n^	RCT
N patients	37 ^a^	138 ^a^	27	24	85
Mean Age	31.6 (SD 7.7)	Not reported	33 (range: 20–70)	44.63 (SD 5.88)	34.2 (SD 10.82)
Female %	75	62.3	48	45.83	68.8
Diagnoses	BPD, AvPD	PD	Mixed ^e^	MDD	MDD
Intervention	Mixed ^b^	CBT, PFPP	Psychoanalytic treatment	STMBP	CBT, IPT
N Therapist	32	24	8	2	34
Duration	65 (SD 60) sessions	19–24 sessions (twice-weekly)	366 sessions (range: 120–2836)	40 sessions	14 sessions
Outcome	SCL-90-R	PDSS	GAF, PHI	GAF, HAM-D	BDI-II
measurement points	T0: baseline	T0: baseline	T1: first 4 sessions	T0: baseline	Before every session
	T1: 8 months	T1: week 1	T2: 4 sessions	T1: 40 weeks (end)	
	T2: 18 months	T2: week 5	after 1 month	T2: 1 year follow-up	
	T3: 36 months	T3: week 10	T3: 1 month before termination		
	T4: 72 months	T4: at termination	T4: last 4 sessions		
Mentalization	RFS (AAI)	RFS (AAI-short ^c^), PSRF (PSRF-I)	CRF (sessions)	RFS (AAI)	RFS, DSRF (AAI-short ^f^)
measurement points	T0: baseline	TO: baseline	T1: first 4 sessions	T0: baseline	T0: baseline
	T1: 36 months	T1: week 5	T2: 4 sessions	T1: 40 weeks (end)	
		T2: at termination	after 1 month	T2: 1 year follow-up	
			T3: 4 sessions in the middle phase		
			T4: 1 month before termination		
			T5: last 4 sessions		
Outcome	Significant improvement T0–T3 (d = 1.0)	Improvement T0–T4	Significant improvement T1–T5	Significant improvement T0–T3	Significant improvement session 1–14 (CBT: d = 1.15; IPT: d = 1.49)
Mentalization change	Not reported	RFS did not improv;e PSRF significantly improved in PFPP, not in CBT	No significant CRF change	Small, non- significant RFS change in therapy (T0-T1); significant RFS change at follow-up (T1-T2)	Not examined
Mentalization-Outcome-relation	a. RFS is not a significant predictor of outcome	Early change in RFS is not significantly associated with outcome change	Early CRF significantly predicts outcome change	RFS significantly predicts outcome change	RFS/DSRF significantly predicts outcome change
	b. RFS is a significant moderator of treatment effects (low RF patients had better outcomes in outpatient individual therapy compared to control condition)	Early change in PSRF was associated with significantly greater change in outcome (the association was stronger for CBT)			
	**Fischer-Kern 2015**	**Karlsson 2006a**	**Karlsson 2006b**	**Müller 2006**	**Taubner 2015**
Publication	[9,47,48,49]	[50,51]	[50,52]	[53]	[18,54]
Country	Austria, Germany	USA	USA	Germany	Germany
Study type	RCT	RCT (archival)	Pre–post (archival)	Pre–post	Pre–post
N patients	92	64	30	24	20
Mean Age	27.7 (SD 7.3); range: 18–51	35 (SD 8.5)	50 (range: 20–81 years)	28 (SD 10)	39.2 (SD 12.7)
Female %	100	70	66.6	70.5	80
Diagnoses	BPD	MDD	Mixed ^i^	Mixed ^j^	MDD
Intervention	TFP, mixed ^g^	CBT, IPT	BPDT	Mixed ^k^	psychoanalytic treatment
N Therapist	67	18	15	not reported	16
Duration	at least 1 year ^h^	16.2 (SD 2.5) sessions	15.8 (SD 1.35) sessions	3 months	227,95 (SD 88,48) hours
Outcome	STIPO	BDI, HSCL-90, HRSD	HSCL-90: GSI, BPRS	SCL-90-R	BDI, SCL-90-R
measurement points	T0: pre-treatment	T1: session 4	T1: session 1	T0: baseline	T0: pre-treatment
	T1: 1 year after start of therapy	T2: session 12	T2: session 5	T1: end of treatment	T1: 24 months in treatment
			T3: session 14		T2: 36 months in treatment
Mentalization	RFS (AAI)	RFS (sessions)	RFS (sessions)	RFS (AAI-shortc)	RFS (AAI)
measurement points	T0: pre-treatment	T1: session 4	T1: session 1	T1: first week in treatment	T0: pre-treatment
	T1: 1 year after start of therapy	T2: session 12	T2: session 5		T1: 24 months in treatment
			T3: session 14		
Outcome	Significant improvement T0–T1	Significant improvement T1–T2	Significant improvement T1–T3	Improvement T0–T1	Significant improvement session 1–14 (GSI: d = 1.64; BDI: d = 2.1)
Mentalization change	RFS significantly improved in TFP, but not in the control condition	Significant RFS decrease (T1–T2)	No significant RFS change	Not examined	Significant RFS increase (T0–T1)
Mentalization-Outcome-relation	RFS improvement significantly predicts outcome change	Process correlates associated with low/high RFS predicted poor/good outcome	RFS is partly related to outcome change	RFS significantly predicts outcome change	RFS significantly predicts outcome change for BDI, but not for GSI
					RFS change had no significant effect on outcome

Note. RCT = randomized controlled trial; BPD = borderline personality disorder; AvPD = avoidant personality disorder; MDD = major depressive disorder; SCL-90-R = SCL-90-R Symptom Checklist-90-Revised; BDI (II) = Beck Depression Inventory; STIPO = structured interview of personality organization; HSCL-90 = Hopkins Symptom Checklist; HRSD = Hamilton Rating Scale for Depression; RFS (*) = Reflective Functioning Scale (obtained from * resource); CRF (*) = Computerized Text Analysis measure of Reflective Functioning [55]; DSRF (*) = Depression-Specific Reflective Functioning; AAI = Adult Attachment Interview; PDSS = Panic Disorder Severity Scale; PSRF = Panic-Specific Reflective Functioning; BPDT = Brief Psychodynamic Therapy; CBT = Cognitive Behavior Therapy; IPT = Interpersonal Psychotherapy; PFPP = Panic-Focused Psychodynamic Psychotherapy, a 24-session, twice-weekly (12 weeks), manualized psychoanalytic psychotherapy [56]; STMBP = short-term psychodynamic psychotherapy with mentalization-based techniques; TFP = Transference-Focused Psychotherapy; ^n^ naturalistc; ^a^ sub-sample of the RCT fulfilling the eligibility criteria; ^b^ mainly psychoanalytic/psychodynamic background, further interventions with cognitive and systemic elements; ^c^ AAI demand questions; ^d^ archival data measured from end of 1960s to 2011; ^e^ depressive disorder, personality disorders, sexual disorder; ^f^ AAI questions 1–11; ^g^ 36.5% psychoanalysis; 34.6% behaviour therapy; 7.7% client-centered therapy, 7.7% systemic psychotherapy, 1.9% gestalt psychotherapy; ^h^ 40.4% of the randomized patients continued therapy; ^I^ depression, dysthymia, and generalized anxiety disorder; ^j^ eating disorders and depressive disorders; ^k^ integrative psychodynamically oriented treatment methods.

**Table 2:** Mentalization scores and changes.

RFS	T1	T2	T1 Control	T1 Control
Antonsen 2016	3.5 (SD 1.7)	*n*/r	3.0 (SD 1.5) ^a^	*n*/r
Bressi 2016	4.00 (SD 2.09)	4.13 (SD 1.80)		
Ekeblad 2016	2.62 (SD 1.22)	*n*/r		
Fischer-Kern 2015	2.82 (SD 1.29)	3.32 (SD 0.99)	2.80 (SD 0.96) ^b^	2.92 (SD 1.00)
Karlsson 2006a	5.13 (SD 1.37)	3.99 (SD 1.35)	3.79 (SD 1.29) ^c^	3.41 (SD 1.26)
Karlsson 2006b	4.62 (SD 1.39)	4.37 (SD 0.82)		
Müller 2006	median: 3 (range: 1–5)	*n*/r		
Taubner 2015	3.85 (SD 0.94)	4.38 (SD 0.93)		
Barber 2020	4.04 (SD 1.23)	4.42 (SD 1.27)	4.39 (SD 1.32) ^d^	4.37 (SD 1.13)
**CRF**	**T1**	**T2**		
Boldrini 2018	29.09 (SD 7.75)	27.25 (SD 9.00)		
**DSRF**	**T1**			
Ekeblad 2016	2.37 (SD 0.98)			
**PSRF**	**T1**	**T2**	**T1 Control**	**T1 Control**
Barber 2020	3.50 (SD 1.19)	4.43 (SD 1.38)	3.68 (SD 1.21) ^d^	3.68 (SD 1.10)

Note. ^a^ outpatient treatment vs. step-down treatment; ^b^ TFP vs. ECT; ^c^ IPT vs. CBT; ^d^ PFPP vs. CBT; *n*/r: not reported.

## Data Availability

The data supporting this systematic review are from previously reported studies, which have been cited. The processed data are available from the corresponding author upon request.
